# Maximum-Entropy Models of Sequenced Immune Repertoires Predict Antigen-Antibody Affinity

**DOI:** 10.1371/journal.pcbi.1004870

**Published:** 2016-04-13

**Authors:** Lorenzo Asti, Guido Uguzzoni, Paolo Marcatili, Andrea Pagnani

**Affiliations:** 1 Dipartimento di Scienze di Base e Applicate per l’Ingegneria, Sapienza University of Roma, Roma, Italy; 2 Human Genetics Foundation, Molecular Biotechnology Center, Torino, Italy; 3 Sorbonne Universités, UPMC, UMR 7238, Computational and Quantitative Biology, 15, rue de l’Ecole de Médecine - BC 1540 - 75006 Paris, France; 4 Dipartimento di Fisica, Universià di Parma, Parma, Italy; 5 Center for Biological Sequence Analysis, Department of Systems Biology, Technical University of Denmark, Lyngby, Denmark; 6 Department of Applied Science and Technologies (DISAT), Politecnico di Torino, Torino, Italy; Bar Ilan University, ISRAEL

## Abstract

The immune system has developed a number of distinct complex mechanisms to shape and control the antibody repertoire. One of these mechanisms, the affinity maturation process, works in an evolutionary-like fashion: after binding to a foreign molecule, the antibody-producing B-cells exhibit a high-frequency mutation rate in the genome region that codes for the antibody active site. Eventually, cells that produce antibodies with higher affinity for their cognate antigen are selected and clonally expanded. Here, we propose a new statistical approach based on maximum entropy modeling in which a scoring function related to the binding affinity of antibodies against a specific antigen is inferred from a sample of sequences of the immune repertoire of an individual. We use our inference strategy to infer a statistical model on a data set obtained by sequencing a fairly large portion of the immune repertoire of an HIV-1 infected patient. The Pearson correlation coefficient between our scoring function and the IC_50_ neutralization titer measured on 30 different antibodies of known sequence is as high as 0.77 (p-value 10^−6^), outperforming other sequence- and structure-based models.

## Author Summary

Affinity maturation is a very complex biological process which enables activated B-cells to produce antibodies with increased affinity for a given antigen. Once B-cells begin to proliferate, each of the progeny cells introduces mutations in the antigen binding region in order to explore different affinities for the antigen. Selection rounds occurring in the so-called *germinal centers* in lymph nodes and spleen prune out poorly binding receptors and clonally expand good binders. Thanks to high-throughput sequencing techniques it is now possible to have access to a fairly representative sample (of the order of 10^5^ to 10^6^ sequences) of the immune repertoire of a given individual. Our approach is to first exploit this large amount of sequence data to infer a statistical model for the sequenced portion of the immune repertoire, and then to use the inferred probability of this model as a score when predicting the neutralization power of a given antibody sequence for the antigen of interest. The results we obtained on a specific data set of sequences of an HIV-1 patient show that our score correlates very well with experimentally assessed neutralization power of specific antibodies of known sequence. The performance of the method crucially relies on the ability of our model to account for long-range intragenic epistatic interactions between residues along the whole antibody chain.

## Introduction

The prediction of antibody (Abs, or immunoglobulins, Igs) affinity for antigens is among the most interesting open challenges across bioinformatics and structural immunology. Most of the current methods rely on the structures (either experimentally resolved or modeled) of both antibodies and their cognate antigens to predict their binding affinity. Currently, available methods are time demanding and, more importantly, their predictions are hard to assess [2, 3]. On the other hand, because of the scarcity of available data-sets for which both Abs sequences and their affinity for an antigen are known, there is still no method that can model the affinity as a function of the sequence of the antibody variable region. Also, it is still not clear if and how it would be possible to set up a coherent fitting procedure to estimate the (possibly) huge number of parameters of a generic mapping from the space of Abs sequences to the affinity for the antigen.

Thanks to the recent developments of sequencing techniques (*e.g.* Deep Sequencing, and Next Generation Sequencing), Repertoire Sequencing (Rep-Seq) experiments (see [4] for a review of the argument) start to be routinely performed. Recently, the complete Ig repertoires of several simple organisms such as the zebra-fish, whose immune system has only ∼300.000 Abs producing B cells, have been sequenced [5]. Higher organisms, such as humans, show a remarkably more complex immune system and it is widely accepted that the typical human Ab repertoire amounts to ∼10^9−10^ different molecules. In this case, a large sample of the entire repertoire can be extracted (see for example [6] for Rep-Seq experiment on Igs in human).

Rep-Seq data allow for a detailed description of the sequences distribution based on Maximum Entropy (MaxEnt) modeling of repertoires, as it has been proven in the case of zebra-fish Abs [7] and human T cell receptors [8, 9]. While these studies focus on a model-based description of the initial repertoire of the adaptive immune system arising mainly from the V(D)J genetic rearrangement, here we focus on the affinity maturation process.

**Fig 1:**
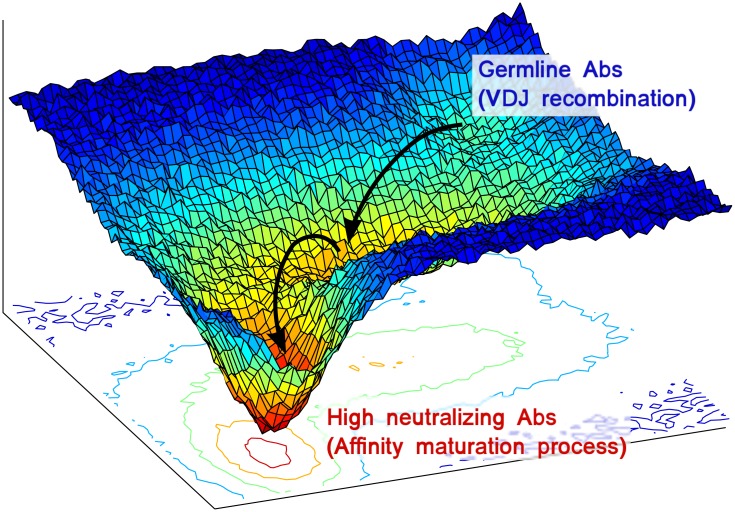
Pictorial representation of the evolutionary dynamics over the fitness landscape in the affinity maturation process.

A number of statistical mechanics inspired methodologies have been recently successfully devised to analyze evolutionarily related proteins for inferring structural properties and, in particular, residue-residue contacts [10]. In particular, homologous proteins can be characterized in terms of multiple sequence alignments (MSAs). In spite of the considerable sequence heterogeneity (up to only 40% sequence identity) in families of homologous proteins, their folded structures are often almost completely conserved [11]. A MaxEnt modeling technique developed more than a decade ago, could detect signals of the evolutionary pressure beyond the sequence variability in MSAs of homologous proteins [12]. Maintaining the same underlying idea that co-evolution of residue pairs is related to their spatial proximity in the folded protein structure, a large number of works successfully reconsidered MaxEnt in different flavors: (i) the application of mean-field approximations known as Direct-Coupling Analysis (DCA) [13–15], (ii) pseudo-likelihood maximization (PlmDCA), [16–18], (iii) Multivariate Gaussian Modeling (MGM), [19, 20]. All these methods rely on the inference of a generative probabilistic model for sequences in the presence of selective pressure. This feature makes this kind of analytic techniques particularly suited for the study of Ab affinity maturation. In fact, this process closely resembles a Darwinian evolutionary framework where B-cell clones compete for the antigen in the germinal centers, and it is now widely accepted that the affinity for the target antigen represents the main contribution to the fitness in this evolutionary scenario. Thus, as qualitatively sketched in Fig 1, for every antigen, the evolutionary dynamics explores the space of Ab sequences searching for the global optimum of the fitness function, i.e. the best affinity for the related antigen.

Here we exploit the evolutionary nature of the affinity maturation process by applying a MaxEnt inference techniques originally developed for the analysis of homologous protein families. The above mentioned plethora of model inference methods aim at reconstructing a reliable contact map from the space of homologous protein sequences through an analysis of residues coevolution that disentangle indirect correlations, but in our context, they provide little information on Abs internal structure. However, the inference procedure provides a natural and reliable scoring function (see Section “Inference Methods”) from the space sequences to that of binding affinity for the target antibody related to the probability for a sequence to appear in the data set that we can use as a proxy to the binding affinity to the antigen, in the spirit of series of recent publications [21–23] where deep sequencing of the immune repertoire was used to predict binding vs. non-binding Abs with different therapeutic applications.

Finally, we report that very recently maximum entropy modeling has been also used in [24] to predict the fitness landscape of the HIV-1 protein from the relative abundance of the virus strains, and in [25] to predict *in silico* the effect of mutations related to disease and antibiotic drug resistance.

## Results

In the present work, we apply MaxEnt methods to study the affinity maturation process on publicly available data from an HIV-1 infected donor [1].

The immune system of this patient had developed over the years the so-called *broadly neutralizing antibodies* (bNAbs), which can bind with high affinity to the virus capsid protein gp120 and impair the viral ability to infect new cells. The broadness of Abs neutralization entails their capability of neutralizing multiple HIV-1 strains, as opposite to non-bNAbs which are specific for individual viral strains. The following data from Wu *et al.*, all derived from the antibody repertoire of the patient, have been used in the present work: (i) a X-ray crystallographic structure of gp120 in complex with VRC-PG04, a broadly neutralizing Ab identified through cell sorting; (ii) a Rep-Seq data of the donor’s immunoglobulins heavy chains (IGH) variable region repertoire (see Section “Deep sequencing data”); (iii) half maximal inhibitory concentration measurements (IC_50_) of chimeric Abs against some isolates of the antigen gp120. IC_50_ will be considered hereafter as a proxy for the IGH contribution to the antigen-Ab complex binding affinity (see Section “Neutralization measurements” for details).

Our study is based on two main working hypotheses: (i) the Ab sequences that are similar to the highly responding Ab VRC-PG04 are informative about their binding energy [1]; (ii) This specific subset of Abs has evolved through affinity maturation, i.e. developing somatic mutations in gp120-binding sequences to enhance their binding energy toward the antigen.

**Fig 2:**
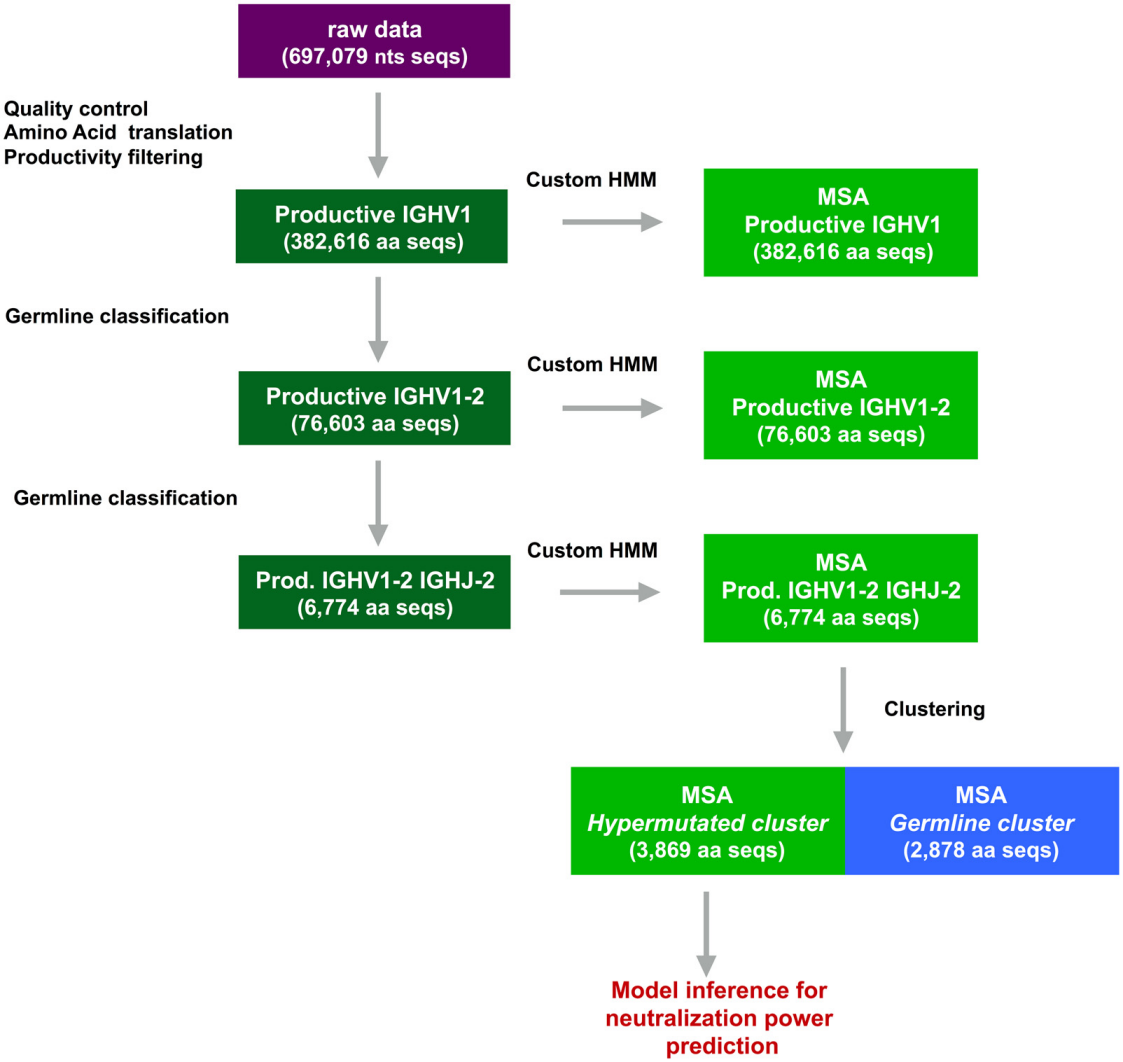
Preliminary bioinformatics analysis. The purple box indicates the raw data set consisting of a 454 pyrosequencing samples of IGH nucleotide (nts) sequences from [1]. Dark green boxes represent sets of sequences that are obtained after the bioinformatics analyses: the first step consists in the identification of primers and conversion to the reverse complement; then, after amino acid translation, non-productive sequences are filtered out; finally, through the use of the IgBLAST software, germline genes of origin are inferred and different subsets of sequences are selected. Light green boxes refer to the corresponding MSA produced through the custom made HMM. The smaller aligned subset is submitted to a clustering procedure that identifies a *germline cluster* and a *hypermutated cluster*. The final MSA is used to infer an MGM model for affinity prediction.

As summarized in Fig 2, we have developed a bioinformatics pipeline to select a subset of aligned Ab amino acid sequences from the whole Rep-Seq data set. We claim that the selected sequences have performed affinity maturation to achieve a high and broad power against gp120. In the “Clustering analysis” section we explain how the choice of the gp120-responding ensemble (which we call from now on *hypermutated cluster*) is done, while in the “Multiple sequence alignments” section we describe how we constructed the custom Hidden Markov model to align sequences.

**Fig 3:**
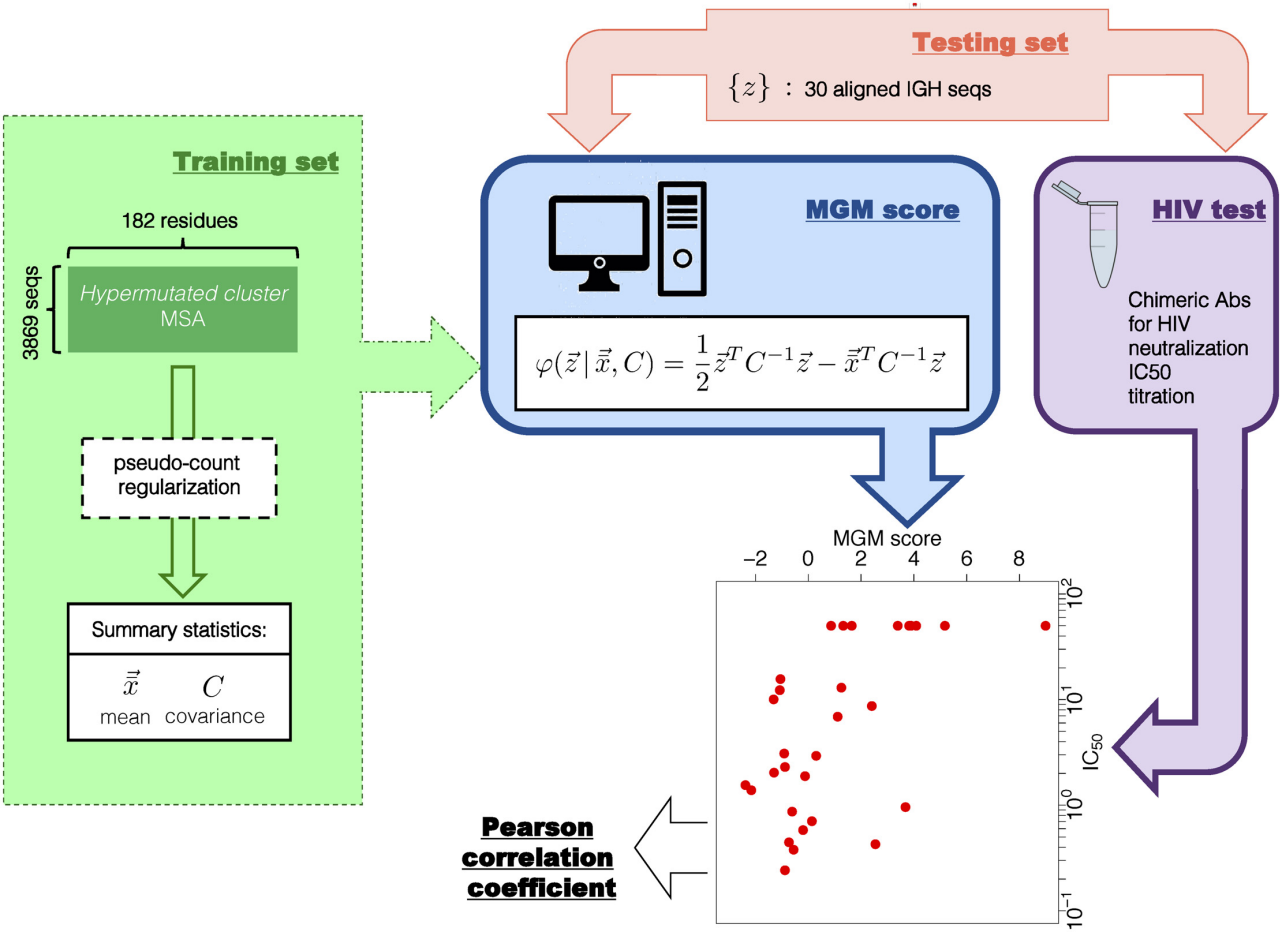
Model inference. We start from the multiple sequence alignment of the heavy chain variable region sequences belonging to the *hypermutated cluster* and define a summary statistics of the data set in terms of the single residue frequency counts (means) $\vec{\bar{x}}$ and the covariance matrix *C* calculated after pseudo-count correction (see [20]). These quantities define the maximum-likelihood MGM, a multivariate Gaussian distribution whose parameters are the mean and the covariance. The exponent of the Gaussian distribution is the MGM-score function ($\vec{z}$ is the amino acid sequence of the Ab to be scored) which is used as a proxy of the binding affinity toward gp120. A set of 30 IGHs is used to test the accuracy of the model in predicting the IGH contribution to the neutralization power. In fact, in [1], these IGHs sequences were used to produce chimeric Abs with the IGL of a known bnAb (VRC-PG04), which were eventually tested for neutralization power against 20 HIV viruses. Here we compare the IC_50_ neutralization titer (averaged over the neutralized viruses) with their MGM-score, as shown in the scatter plot, where the choice of the pseudo-count parameter is *π* = 0.2. The performance of the prediction is eventually assessed in terms of the Pearson correlation coefficient between the two quantities.

From these premises we used MGM [20] (see Section “Multivariate Gaussian Modeling”), a particular version of MaxEnt modeling, to infer an accurate statistical model for the ensemble of Abs in the data set clonally expanded for their affinity against antigen gp120, as schematically shown in Fig 3. The MGM model allows taking into account in a probabilistic sense long range intragenic epistatic interactions across the whole heavy-chain variable region of the Ab. Furthermore, the inferred model naturally defines a statistical scoring function (MGM-score) for Ab sequences. In Section “Affinity predictions” we show that the MGM-score correlates significantly (Pearson correlation coefficient up to 0.77) with the IC_50_ assay performed on a large set of Abs of known sequence. We stress that: (i) the MGM-score is inferred on the *hypermutated* set of sequences for which IC_50_ measurements are not available; (ii) the set of artificial chimeric Abs of VRC01 origin (a human immunoglobulin that neutralizes about 90% of HIV-1 isolates) for which the IC_50_ measures are available were not part of the data set from which the MGM was inferred.

We further investigated whether the intragenic epistatic signal captured by the MGM is related to the structural properties of the gp120-Ab complex. In Section “Structural predictions” we discuss our findings: even if the DCA score [20] is poorly correlated with the internal structure of the Ab (as shown in Section “Contact map predictions”), we find a weak signal that can be used in combination with IC_50_ measurements to predict residues that are part of the interaction surface (as shown in Section “Prediction of binding sites”).

### Affinity predictions

Wu and coworkers [1] used 70 sequenced heavy chain variable regions, which originated mostly from immunoglobulins using the IGHV1-2 gene, for constructing chimeric antibodies by combining them with the light chain of VRC-PG04. Among these, 45 have been tested for their neutralization power against 20 HIV-1 mutations.

When included in the sequencing data set and used as input for the clustering procedure, 30 of these 45 tested Abs are found to belong to the *hypermutated cluster*. The remaining 15 (none of which was found to be neutralizing) belong to the *germline cluster*. Although in general the neutralization power depends on both the light and the heavy chain sequences (cf. Fig. 4A in [1]), the light chain plays only a minor role in the interaction (most notably steric contacts with its CDR1 and CDR3 regions) here, as visible from the solved structure of VCR-PG04 (PDB code 3SE9). We therefore will make the simplifying assumption that the neutralization measurements on chimeric Abs depend on the heavy chain contribution alone.

Under the assumption that the *hypermutated cluster* is a statistically representative sample of the Abs that underwent affinity maturation against gp120, we can use the statistical properties of this set of sequences to construct a predictor for the Abs neutralization power. We thus inferred an MGM on the MSA of this cluster and used the MGM-score of the inferred model as a proxy for the neutralization power of the related Abs. Although the inference step is completely blind to the binding affinity of the Abs (the binding affinities of sequences belonging to the *hypermutated cluster* were not measured in [1]), nonetheless the capability of predicting binding energies is not unexpected. Indeed, the aim of a maximum entropy model of the hypermutated set, is to provide an accurate statistical description of the set of Abs responding to gp120, and so it is not completely surprising that, according to the model, sequences with low probability are more likely to have a low binding affinity for the antigen compared to sequences of high probability.

To test the predictive power of the method, we used the panel of 30 sequences (not included in the *hypermutated cluster*) tested for HIV neutralization power and compared the IC_50_ neutralization titer with the MGM-score of the same sequence. Note that values of IC_50_ that are reported in [1] as greater than 50 *μ*g/ml (not-neutralizing) are considered here to be equal to this value. The two quantities are compared by means of the Pearson correlation coefficient. We consider as measures of the neutralization power the average IC_50_ over the different neutralized viruses. A scheme of the model inference and testing procedure is shown in Fig 3.

**Fig 4:**
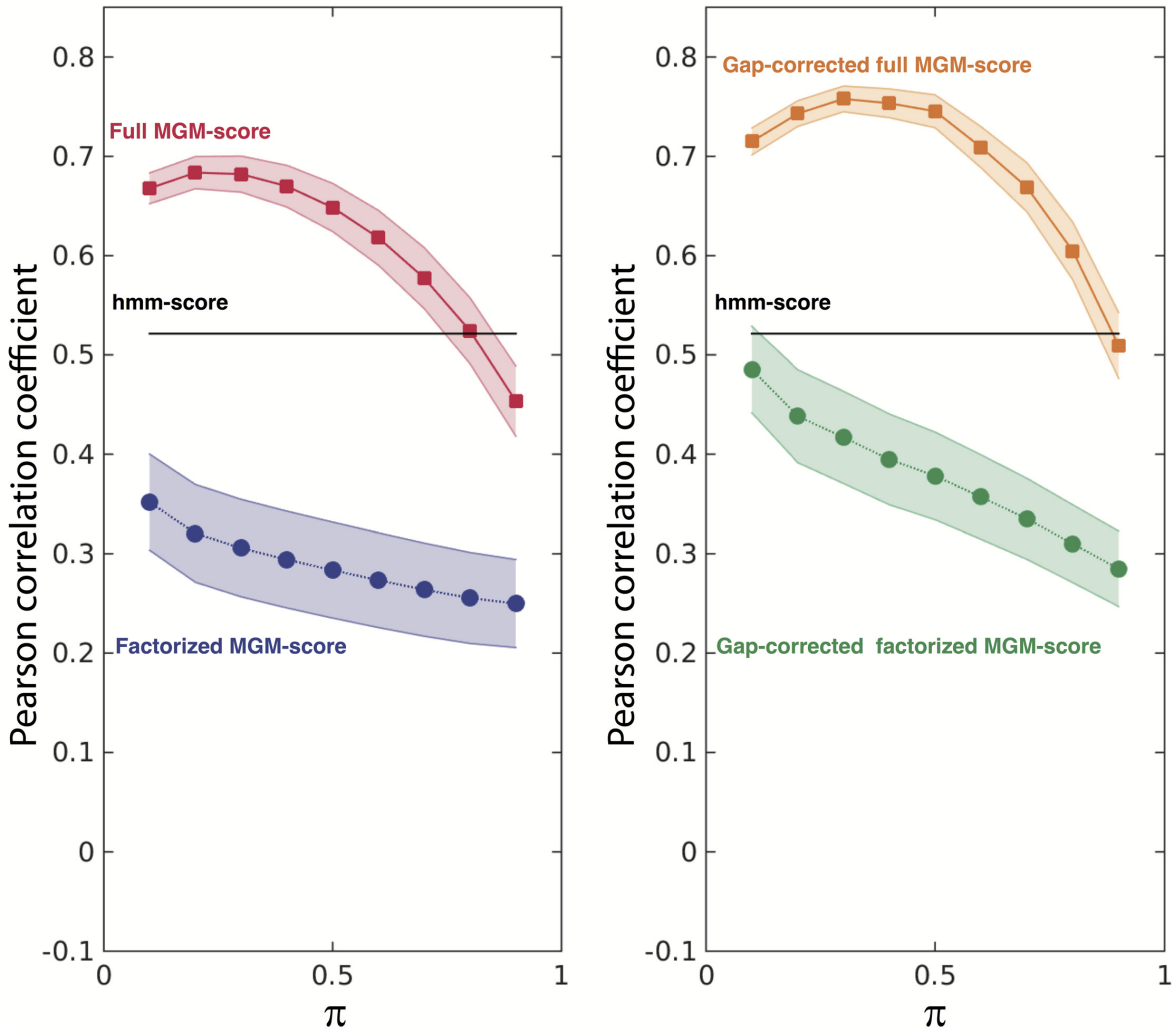
Pearson correlation coefficient between the inferred MGM-score and the average IC50 neutralization titer measured over the 30 tested Abs as a function of the pseudo-count parameter *π* (see Section Inference Methods). For each Ab, the average IC_50_ is computed over the neutralized viruses (IC_50_ < 50*μ*g/ml). Full MGM-score is represented by square bullets joined by continuous lines. Factorized MGM-score is represented by circular bullets joined by dashed lines. The continuous black line shows the correlation value achieved using the hmm-score as an affinity predictor. Error bands are computed with a standard jack-knife re-sampling procedure. *Left panel*: MGM-score. *Right panel*: Gap-corrected MGM-score.

**Fig 5:**
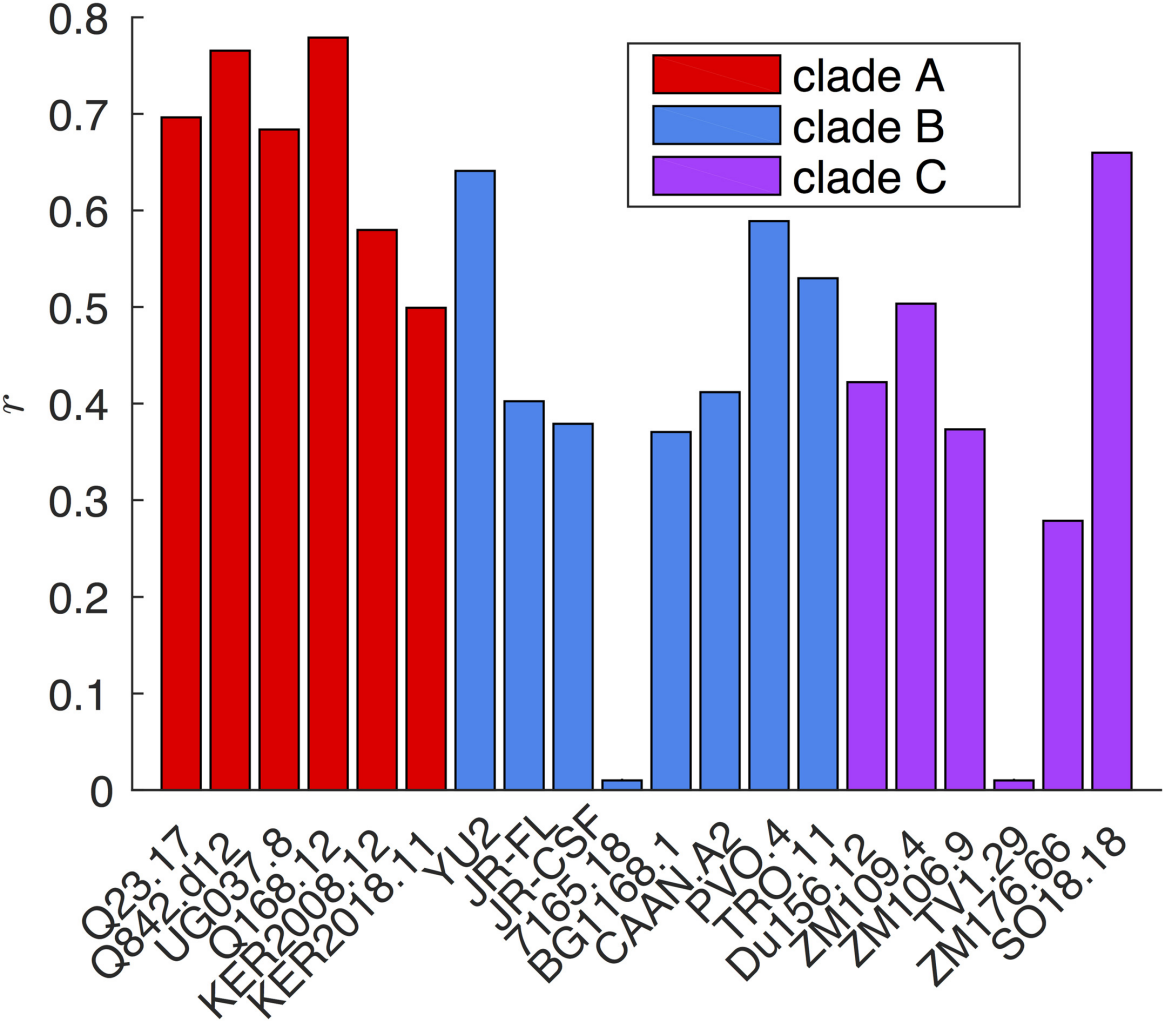
Pearson correlation coefficient *r* between the gap-corrected MGM-score (pseudo-count *π* = 0.3) computed over the 30 sequences in the *hypermutated cluster* and the neutralization power against 20 tested viral isolates belonging to clades A, B and C. The HIV-1 isolates belonging to clade A display a more pronounced correlation. This is consistent with the fact that the donor is known to be infected with an A/D recombinant virus. Note that the poor performance resulting for viruses 7165.18 and TV1.29 are expected since in the experimental assay both viruses were not neutralized by any of the tested Abs (*i.e.* IC_50_ > 50 *μ*g/ml).

The result of the model inference procedure depends on the choice of the regularization parameter *π* defined in the “Inference methods” section. We therefore repeated the test procedure for different values of *π*. In Fig 4 the Pearson correlation coefficient between the MGM-score and the average IC_50_ over the neutralized viral isolates is shown for different values of *π*. The two panels refer to the two score proposed: the original inferred MGM-score and the MGM-score with gap correction (see Section “Score with gap correction” for details). We thus argue that the MGM-score inferred on a representative Rep-Seq data set provides a remarkably good proxy for the neutralization power of the analyzed sequence. We also display the details of our best performance on a per-virus base in Fig 5.

We also assessed the performance of the MGM-score to discriminate binding vs. non-binding sequences. The dataset in this somehow simpler task reduces in a set of 21 non-binding and 24 binding sequences. The performance of the MGM-score are displayed in terms of the ROC curve shown in Fig. F in S1 Text (red curve): the (normalized) area under the ROC (AUROC) turns out to be 0.97. We also compared this value against a much simpler scoring strategy defined in terms of the Hamming distance from the consensus sequence of the hypermutated cluster. As shown in Fig. F in S1 Text (blue curve), the AUROC turns out to be 0.86.

We also inferred the model using PlmDCA [17] rather than MGM. The results are shown in Fig. G in S1 Text: The best Pearson correlation coefficient obtained with this method of inference is slightly worse than the one obtained with MGM. This result is non-trivial since PlmDCA is known to perform better than MGM in terms of protein contact prediction. We also note that in a recent publication [25], a variant of DCA (mean-field DCA) that is essentially equivalent to MGM was used to successfully predict the ΔΔ*G* between mutants and wild type sequences for the beta-lactamase TEM-1.

A natural question is whether simpler inference strategies might achieve equally good results, and in particular whether it is necessary to use the second order statistics (*i.e.* multivariate *vs* univariate statistics) to infer Abs neutralization power. To this end, we tested a simpler version of the model, *factorized* over the different residues of the MSA. In this model the non-diagonal *J* terms are set to zero so that the residues are statistically independent (see Section “Multivariate Gaussian Modeling” and [20]). As shown in Fig 4 (squares and dashed lines), the Pearson correlation coefficient is dramatically reduced, dropping from a maximum of 0.77 for the full MGM to a maximum of 0.49 for the factorized model.

Our neutralization power predictor was compared with another sequence based method, the HMM-score (see Section “Using Hidden Markov Models to predict binding affinities”). This score takes only correlations between nearest neighbors in the sequence into account. Interestingly, as displayed in Fig 4, the prediction quality of this method is between the one obtained using the factorized MGM-score and the one obtained using the full MGM-score. This supports the observation that long range intragenic epistatic signals are crucial to reproduce neutralization power.

An important step in the procedure is to correctly identify the set of sequences that underwent affinity maturation towards the same epitope. Indeed, MGM models trained on different sets (for example the entire set of sequences coming from the germline of interest) display no significant correlation with neutralization measurement.

Some portions of the MSA are observed to be more important than others in reproducing the affinity function: The correlation between the inferred likelihood and the neutralization titers is essentially the same when only the ∼60 more variable residues of the *hypermutated cluster* MSA are used to construct the MGM, dismissing ∼3/4 of the columns of the MSA. Data of this MSA reduction analysis are reported in S1 Text (see Section “Affinity predictions”).

Our predictor was also compared with a structure-based method: we produced structural models for all the 45 antibody/antigen complexes for which the IC_50_ was measured and predicted their binding affinity using FoldX (see Methods for details). The results of this structural method show no significant correlation (*r* = −0.23, p-value = 0.13) with the experimental data.

Taken together, our findings indicate that: (i) MGM inferred on the proper set of clonally expanded sequences contains enough information to predict the neutralization power of Ab sequences. This suggests that the procedure can be used as a tool to generate new and highly neutralizing Abs; (ii) taking into account (pairwise) intragenic epistatic effects in the model improves remarkably the accuracy of the affinity prediction.

### Structural predictions

#### Contact map prediction

In the previous Section, the accuracy of the statistical model as a neutralization power predictor has been assessed. We now analyze the performance of MGM modeling in predicting pairs of residues which are in contact assuming that the structure of Abs in the set can be approximated with that of VRC-PG04. The structure of Antibodies is known and very well conserved, so the main aim of this test is about the nature of the affinity maturation process which besides selecting Abs with high affinity with the antigen, must also produce structurally stable proteins.

**Fig 6:**
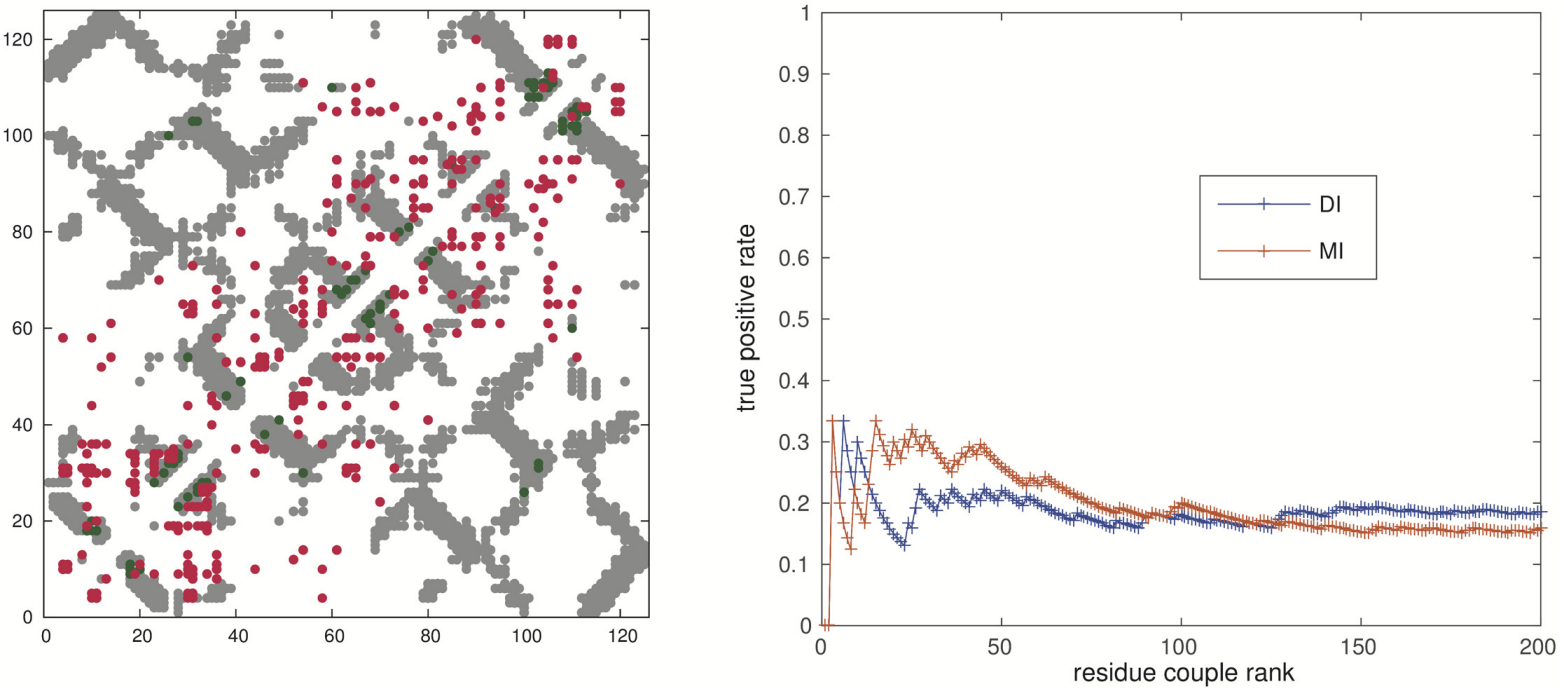
*Left Panel:* Direct Information map computed on the *hypermutated cluster*. The internal contact map of the VRC-PG04 heavy chain is shown in gray (PDB 3SE9). Two residues are considered to be in contact if at least a pair of heavy atoms is at a distance lower than 8Å. The first 300 couples with higher Direct Information DI [13] are displayed in green when they superpose to the internal contacts (true positives internal contact predictions) and in red when they do not (false positive internal contact predictions). *Right Panel:* Sensitivity plot of the Direct Information (DI) and Mutual Information (MI).

For these reasons, we first inferred an MGM on the *hypermutated cluster*, and then used the Direct Information (DI) between residues as a predictor for contacts (see [20]). The results are presented in Fig 6. It can be seen that MGM modeling is not able to capture relevant structural information. One may wonder if the sequence variability in the *hypermutated cluster* is not enough for detecting structural information. To check this hypothesis we performed the MGM inference over the set of all sequenced reads independently of the germline genes of origin. The internal contact prediction is only marginally improved as discussed in S1 Text (see Section “Internal contacts”). Qualitatively similar results are obtained using PlmDCA [17]. We speculate that the timescale over which affinity maturation occurs is too short when compared to the time scale that separates evolutionarily related proteins in protein families. Therefore, the sequence space explored by the Abs repertoire is not large enough to generate significant statistical correlations due to internal contacts. Of course, we cannot exclude that our method simply fails to detect weak evolutionary signals.

#### Predictions of binding sites

In the previous Section, we have shown that the statistical properties of the Rep-Seq data do not seem to provide information on the Ab structure. Nevertheless, we now show that the combination of the statistical properties and the neutralization power measurements allows recovering some structural information about the antigen recognition mechanism.

If the number of residues of the MSA is progressively reduced by eliminating residues of the MSA with decreasing entropy (i.e. variability), the correlation between the inferred MGM-score and the neutralization power is progressively reduced. We have analyzed the position in the crystallographic structure of all the residues which, upon removal of the corresponding column from the MSA, lead to the sharp decays in the correlation value. The results are resumed in Table 1, while the corresponding plots are reported in S1 Text (see Section “Ab-antigen interactions”).

**Table 1:** Prediction of Ab-gp120 binding sites classified as *binding* if the minimum distance between any atom of the residue and the antigen is less than 5Å, *proximal* if the distance is between 5Å and 10Å, *distant* if it is more than 10Å. Note that the PDB structure 3SE9 shows 17 *binding* residues in the VRC-PG04 heavy chain which is 225 residues long.

3SE9 Chain H	binding role
A 16	distant
E 26	proximal
D 27	distant
F 91	distant
R 73	binding
S 68	proximal
E 33	proximal
V 110	distant
H 35A	binding

Most of the highlighted residues (apart from amino acid A 16) are mutated from the germline in the PDB structure 3SE9 (chain H) (of course all residues are mutated in at least a few sequencing reads). The only residue that actively binds to the antigen is R 73. However, many of them (the ones marked as *proximal* in Table 1) are in the so-called *Vernier zone* for this antibody. This means that they are in contact with residues that bind the antigen and therefore potentially have a role in the interaction by influencing the local environment. This analysis therefore retrieves information about the antigen recognition mode. It can generally be applied when the 3D structure of the Ab-antigen complex is lacking but Rep-Seq data and neutralization measurements are available.

## Methods

### Data

#### Deep sequencing data and bioinformatics pipeline

The Rep-Seq experiment performed on a Roche 454 pyrosequencing platform in [1] aimed to study mutations in the variable regions of both heavy and light chains of the Igs repertoire of the donor. Unfortunately, light and heavy chains are translated into different mRNA molecules. As a consequence, the sequencing technique captures the mRNA in the sample and different mRNA molecules belonging to different cells are mixed during the procedure. Therefore, the light and heavy chain repertoires are only separately available and there is no way to reconstruct the entire antibody sequence.

As summarized in Fig 2, after having performed a standard sequencing data analysis and a quality control, we identified all the sequences likely to belong to bnAbs. These antibodies have been hypothesized [1] to have matured from subsequent expansions of an original clone expressing the IGHV1-2*01 and IGHJ*02 germline genes—see [26] for information on (IMunoGenTics, IMGT) IGH genes nomenclature scheme. Therefore, in order to identify the ensemble of bnAbs from the whole donor Ab repertoire, we used the IgBLAST platform [27] to assess both germline gene of origin and productivity. We first selected sequences with productive amino acid translation and then, in a subsequent step, we screened sequences of those Abs that mutated from these particular germline genes.

Data are available from National Center for Biotechnology Information Short Reads Archives (SRA) under accession number SRP006992.

#### Multiple sequence alignments

Ab sequences have been aligned by taking advantage of a custom Hidden Markov Model (HMM). We first aligned our data set according to the Kabat-Chothia numbering scheme, using a modified version of the antibody-specific HMMs developed by us previously [28, 29]. The first modification, following the IMGT [30] and AHo [31] numbering schemes, was to place the H3 insertions symmetrically in the central position between residue 94 and 101, thus obtaining a better alignment of the H3 regions neighboring the loop stems.

A second modification to the HMM was needed since a large fraction of the antibodies in our data set has diverged considerably from their original germline sequence. This gives rise to insertions and deletions that are uncommon in the normal antibody repertoire and that resulted in sequences with a poor alignment score in the H2 region. The problem could be solved by adding an insertion between residue 59 and 60 in the Kabat-Chothia numbering scheme. A posteriori, this insertion was confirmed by the analysis of the solved structure of VRC-PG04 in complex with gp120 (PDB code 3SE9), in which the insertion is located at residue at position 59 of the heavy chain following the original PDB file numbering. The same position corresponds to an insertion between residue 59 and 60 in the Kabat-Chothia numbering scheme. Accordingly, we modified the alignment originally used to generate the HMMs by introducing such insertions and used them to produce the final multiple sequence alignments, whose characteristics are resumed in Table 2.

**Table 2:** Summary of the Rep-Seq data.

set description	size	size (unique)	MSA length
Productive IGHV1 origin	382116	190762	606
Productive IGHV1-2 origin	72603	37793	396
Productive IGHV1-2 and IGHJ2 origin	6774	3212	215
Productive IGHV1-2 and IGHJ2 origin—*germline cluster*	2878	1634	193
Productive IGHV1-2 and IGHJ2 origin—*hypermutated cluster*	3896	1578	182

#### Crystallographic structure

The crystallographic structure of the broadly neutralizing antibody VRC-PG04 in complex with gp120 described in [1] is available in the Protein Data Bank under the identification 3SE9.

#### Neutralization measurements

The neutralization power of 45 chimeric Abs, in which VRC-PG04 light chain was coupled with heavy chains selected from the highly mutated (more than 25% divergent from the IGHV germline gene) ones in the sequenced set was measured in [1].

As a result of the neutralization measurements on 20 HIV-1 isolates belonging to the clades A (6 viruses), B (8 viruses) and C (6 viruses), it turns out that heavy chains that are more similar to VRC-PG04 are in general more broadly neutralizing (see Fig. 4 in [1]).

### Clustering analysis

The identity/divergence analysis performed in [1] on the whole deep sequencing data set indicates that sequences with inferred IGHV1-2 germline gene (the same of VRC-PG04) are characterized by: (i) the presence of a cluster of highly mutated sequences that is well separated from the cluster of typically mutated sequences; (ii) Abs with a different IGHV inferred germline gene display a more uniform (i.e. less *clustered*) structure.

**Fig 7:**
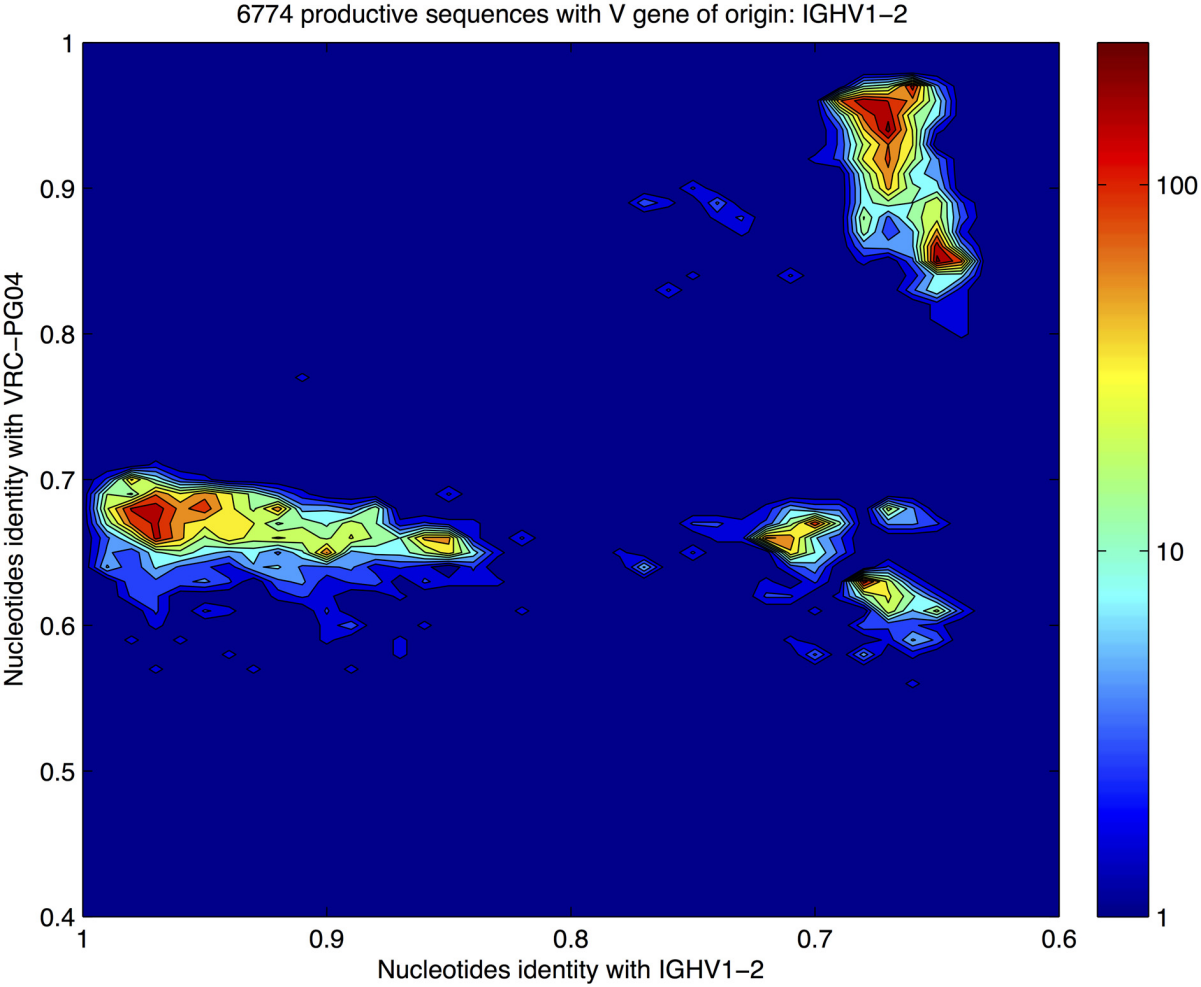
Density plot of the identity/divergence analysis performed on productive sequences with inferred germline IGHV1-2 gene. Identity with the IGHV1-2 gene and with the bnAb VRC-PG04 nucleotide sequences are reported respectively on the horizontal and vertical axes. We identify the high-density zone in the upper right zone of the plot (large divergence from the germline gene IGHV1-2 and similar to the bnAb VRC-PG04) with the *hypermutated cluster* of sequences clonally expanded to respond to gp120.

We performed an independent identity/divergence analysis on the data set resulting from our bioinformatics analysis in which we retain only productive sequences of IGHV1-2 origin. Our results are in complete agreement with [1], as shown in Fig 7. There we compare the identity to VRC-PG04 and the divergence from IGHV1-2*02 germline gene at a nucleotide level for each sequence in the data set.

Identity/divergence analysis gives a glimpse of the structure of the sample in the space of sequences. Nevertheless, a less biased analysis is required in order to test the cluster structure. We thus performed a sequence-based clustering analysis. Among the different clustering algorithms available, we chose the *shallow tree clustering algorithm* [32] since it provides a criterion of robustness against noise (see S1 Text Section “Sequence clustering analysis”). The clustering algorithm is based on the Hamming distance between sequences.

The most robust solution (see S1 Text for an explanation of what robust means in this context) found by the algorithm is a partition of the sequences into two clusters: a *germline cluster* composed of 2878 sequences (1634 unique) centered on the IGHV1-2*02 and IGHJ2*02 germline genes (with an average sequence divergence of ∼5% from the germline), and a *hypermutated cluster* composed of 3896 sequences (1578 unique) more similar to the broadly neutralizing antibody VRC-PG04 (with an average sequence divergence of ∼35% from the germline, see Section “Clustering analysis” in S1 Text for details). These results are confirmed by a test with the *k-means* clustering algorithm (run with *k* = 2). Information about the two clusters and their MSA characteristics are resumed in Table 2.

In the present work, we assume the *hypermutated cluster* to be a representative sample of the Abs that underwent affinity maturation for neutralizing HIV-1 gp120.

### Inference Methods

#### Multivariate Gaussian modeling

Here we define the notation used in the Multivariate Gaussian Modeling. More details can be found in [20].

An MSA of the (horizontal) length *L* of *M* sequences is represented by a *M* × *L*—dimensional array $A\;={\left(a_{i}^{m}\right)}_{\;i\;=\;1,\;\ldots ,\;L}^{\;m\;=\;1,\;\ldots ,\;M}$. Here, *a* belongs to an alphabet of *Q* + 1 = 21 symbols corresponding to the *Q* = 20 natural amino acids plus the “gap” symbol (-).

The MSA is transformed into a *M* × (*Q* ⋅ *L*)—dimensional array $X={\left(x_{i}^{m}\right)}_{\;i\;=\;1,\;\ldots ,\;QL}^{\;m\;=\;1,\;\ldots ,\;M}$ over a binary alphabet {0, 1}. More precisely, each residue position in the original alignment is mapped to *Q* binary variables, each one associated with one of the twenty standard amino acids, taking value one if the amino acid is present in the alignment, and zero if it is absent; the gap is represented by *Q* zeros (i.e. no amino acid is present). Consequently, at most one of the *Q* variables can be equal to one for a given residue position. Thus, for each sequence, the new variables are collected in one row vector, i.e. $x_{(l-1)Q+a}^{m}=\delta_{a,a_{l}^{m}}$ for *l* = 1, …, *L* and *a* = 1, …, *Q*. The Kronecker symbol *δ*_*a*, *b*_ equals one for *a* = *b*, and zero otherwise.

Denoting the row length of *X* as *N* = *QL*, we introduce the empirical mean $\bar{x}$ and the empirical covariance matrix *C*(*X*):

$$\begin{array}{ccc} {\bar{x}}_{i} & = & \frac{1}{M}\sum_{m=1}^{M}x_{i}^{m}\;, \end{array}$$ (1)

$$\begin{array}{ccc} C_{ij}\left(X\right) & = & \frac{1}{M}\sum_{m=1}^{M}\left(x_{i}^{m}-{\bar{x}}_{i}\right)\left(x_{j}^{m}-{\bar{x}}_{j}\right)\;. \end{array}$$ (2)

In order to be inverted, the covariance matrix needs to have full rank. As the region of the sequence space sampled in an MSA is generally limited, the experimental covariance matrix is usually rank deficient. To overcome this problem a regularization procedure has to be implemented. The simplest solution is to add to the sample a number *λ* of fictitious sequences in which amino acids at every site are drawn from a flat distribution. This means to introduce change the frequencies

$$\begin{array}{ccc} {\bar{x}}_{i} & ⟶ & \left(1-\pi \right)\;{\bar{x}}_{i}+\pi \frac{1}{q}\;, \end{array}$$ (3)

$$\begin{array}{ccc} C_{ij} & ⟶ & \left(1-\pi \right)\;C_{ij}+\pi \frac{1}{q^{2}}\;, \end{array}$$ (4)

where the parameter

$$\pi \equiv \frac{\lambda }{M+\lambda }\;,$$ (5),

which is referred to as the pseudo-count parameter, interpolates between the empirical (*π* = 0) and completely random (*π* = 1) data.

The multivariate Gaussian distribution of a set $\vec{x}={\left(x_{i}\right)}_{\;i\;=\;1,\;\ldots ,\;N}$ of variables, is parametrized by a mean vector $\vec{\mu }={\left(\mu_{i}\right)}_{\;i\;=\;1,\;\ldots ,\;N}$ and a covariance matrix as Σ = (Σ_*ij*_)_*i*, *j* = 1, …, *N*_ as:

PG(x→|μ→,Σ)=1(2π)NdetΣexp-12(x→-μ→)TΣ-1(x→-μ→)∝exp-E(x→|μ→,Σ). (6)

The exponent *E* in the previous Equation which is usually parametrized as:

E(x→|μ¯→,Σ)=12(x→-μ→)TΣ-1(x→-μ→)==-12x→TJx→-h→Tx→, (7)

where *J* = −Σ^−1^ is called the *interaction matrix* (*precision matrix* in the probability theory language) and $\vec{h}=C^{-1}\vec{\mu }$ are indicated as the *external fields*.

In the following, we will refer to the latter model as the *full* MGM model. A simpler, yet interesting case is given by a factorized Gauss distribution, for which the MGM-score is still given by Eq (6) but Σ is now block-diagonal (i.e. the probability is a product of independent Gaussian for each residue in the alignment). We will refer to this second model as the *factorized* MGM model.

Having now measured and corrected $\vec{\bar{x}}$ and *C* following Eqs (1), (2), (3) and (4), the maximum likelihood estimate of the probability density function of a given sequence $\vec{z}$ is

$$P_{G}^{ML}\left(\vec{z}\;|\;\vec{\bar{x}},C\right)=P_{G}\left(\vec{z}\;|\;\vec{\mu }=\vec{\bar{x}},\Sigma =C\right)\;,$$ (8)

so that

$$\begin{array}{ccc} J_{ML} & = & -C^{-1}\;, \end{array}$$ (9)

$$\begin{array}{ccc} {\vec{h}}_{ML} & = & C^{-1}\vec{\bar{x}}\;. \end{array}$$ (10)

The MGM-score is defined as the maximum likelihood estimate of the exponent defined in Eq (7) as:

$$\begin{array}{ccc} \varphi (\vec{z}\;|\;\vec{\bar{x}},C) & = & -\frac{1}{2}{\vec{z}}^{T}J_{ML}\vec{z}-{\vec{h}}_{ML}^{T}\vec{z}\;=\\ & = & \frac{1}{2}{\vec{z}}^{T}C^{-1}\vec{z}-{\vec{\bar{x}}}^{T}C^{-1}\vec{z}\;. \end{array}$$ (11)

Given an MSA, a standard measure of the correlations between the amino acid usage at different positions in the alignment is given by the Mutual Information (MI). As all correlation measures, the MI does not distinguish between *direct* and *indirect* correlations, i. e. between correlations that have a direct or indirect relationship. In distinction to such measures, the inferred maximum likelihood probability distribution Eq (8) is a quantity that contains information about the statistical behavior of the whole set of variables and not only of a pair of them (as in the case of MI). Statistical interactions are thus only direct and, in our framework, they are encoded in the interaction matrix *J*. As *J* is a *QL* × *QL* matrix and we want a numeric measure of the (statistical) interaction between two sites (columns in the MSA), we need to associate a single scalar score to each *Q* × *Q* block in the matrix. This can do coherently by computing the so-called *Direct Information* (DI) map from the inferred *J* and *h*, which is a *L* × *L* matrix encoding interaction scores between couples of columns in the MSA. A more detailed description of the previous formula and of DI map can be found in [20].

#### Score with gap correction

A known pathology of MSAs of highly heterogeneous sequences is that the statistical properties of gaps are different from those of ordinary residues (see [33] for a discussion of this problem in the context of the contact map prediction). This phenomenon is known to produce spurious correlations between residues in the alignment that can affect the performance of inference, in particular in the under-sampling regime. To deal with this problem, we introduce a procedure to lower the influence of gaps on the MGM-score: in each sequence, gaps are maintained and amino acid symbols are randomly replaced by the background amino acid distribution computed over the whole alignment. This procedure aims at obtaining a null-model alignment that maintains only the correlations due to gaps.

From both alignments (null-model and original), we define a gap-corrected MGM-score as the difference between the MGM-scores computed from the two alignments. The improvement in the prediction of the binding affinities of the original score *vs* the modified one is shown in Fig 4. In the right panel we display the Pearson correlation coefficient when we use the gap-corrected MGM-score, and in the left panel the same with the original MGM-score.

### Structural analysis

The structure of VRC-PG04 in complex with gp120 (PDB-id 3SE9) has been subjected to both visual inspection and quantitative predictions to assess the importance of each somatic mutation observed in the antibody to the binding affinity towards the antigen. Somatic mutations were retrieved using the IMGT database [34]. We used the FoldX software [35] to predict the difference in binding energy (ΔΔG) of the actual antibody with all the mutants obtained reverting each single somatic mutation to the original residue observed in the germline gene IGHV1-2.

### Alternative methods to infer binding affinities

#### Structural prediction of the binding affinity

In order to compare the results obtained with our sequence-based method to some structure-based predictions, we modeled all 45 antibodies for which the affinity was measured. We used the HMM explained above to align all the heavy chain sequences to the heavy chain of 3SE9; such alignments were then used as input for Modeler (v9.12) [36] to build all the models using 3SE9 as a template and the option md_level = refine.fast. This was done to fix possible differences in loop length and physico-chemical errors introduced by the homology modeling. FoldX was subsequently used on each model to evaluate the interaction energy between the antibody and the antigen and these predictions were eventually compared with the experimental values reported in the original paper.

#### Using hidden Markov Models to predict binding affinities

In order to compare the results obtained by MGM modeling with another sequence-based technique, we used a Hidden Markov Model based strategy based on the HMMER suite (v3.1b2) [37, 38]. From the multiple sequence alignment built on the set of sequences belonging to the *hypermutated cluster*, we first extracted the HMM with the command hmmbuild. We then used the program hmmsearch to produce a score for each of the 45 Abs. All programs were run with default parameters. There are two different scores produced by hmmsearch: the E-value (we considered the negative log transform of this quantity) and the so-called hmm-score. The Pearson correlation coefficient of these two scores with the measured IC_50_ is the same within error bars. For this reason, we will only report the correlation with the hmm-score.

#### Using different inference algorithms

The last step of the pipeline showed in Fig 2 is the inference of the Maximum Entropy models from the MSA statistics. Other MaxEnt methods can be used for the same sake. In particular, we compared the performance of MGM with that of pseudo-likelihood maximization method (plmDCA), an approximated algorithm for MaxEnt inference. This method is widely used in the context of sequence-based structure predictions in proteins, due to the better performance in recovering the internal contact maps. We computed the plmDCA model parameters from the *hypermutated cluster* MSA and defined the score as the log-probability of an Abs sequence. The plmDCA score is less effective in reproducing affinity measures then the MGM-score. However, it has as expected a better performance than the factorized MGM-score and the hmm-score, as shown in Fig. G in S1 Text.

## Discussion

In the present study, we proposed a sequence based maximum entropy model to analyze Ab affinity for the antigen. The predictive validity of the model has been tested using Rep-Seq data and neutralization power measurements from an HIV-1 infected donor [1]. The interplay between the HIV-1 virus and the immune response provides an interesting framework for our purpose: the affinity maturation of the Abs of interest (those whose epitope is the gp120 CD4 binding site) causes a dramatic increase of their neutralization power and a pronounced mutation ratio in comparison with the germline genes. This high density of mutations allows us to easily select sequences in the immune repertoire that respond to the antigen.

A maximum entropy model constructed on this set of hypermutated sequences has been successfully used as a predictor of the neutralization power of Abs. This predictor has been successfully assessed against experimental neutralization measurements of different viral isolates. These positive results suggest that the procedure could be used as a tool for generating new and highly neutralizing Abs.

In analogy with the application to protein families [12–20], the MaxEnt model has been used for predicting residue-residue contacts in the Rep-Seq sample without obtaining positive results. This is not surprising since the time-scale involved in the affinity maturation process (years) is not comparable to the typical evolutionary time-scale in protein families (millions of years).

The structure of the inferred statistical interactions is probably mostly driven by the interaction with the epitope and further investigations in this sense represent an interesting development of this work. Nevertheless, the joint analysis of the sequencing data statistics and neutralization measurements has been shown to provide some consistent structural information on antigen recognition mode.

In conclusion, the use of maximum entropy models can unveil relevant features of the protein fitness function. These features are related to the affinity maturation process and in particular to the evolutionary dynamics of the B cell population. This could be of interest for a statistical population genetics analysis of the affinity maturation process (for example in the spirit of [39] and [40]). The present case study shows how MaxEnt methods can be a useful tool for tackling immunological questions in a time when Rep-Seq data are becoming increasingly popular in immunology (see for instance [41], where T receptor repertoires are studied).

## Supporting Information

S1 Text Supplementary Methods and Results. Methods: Preliminary deep sequencing data analysis, multiple sequence alignment, clustering analysis, Supplementary results: Affinity predictions, structural predictions, internal contacts, ab-antigen interactions.
(PDF)
