# Supplementary material for: Maximum-Entropy Models of Sequenced Immune Repertoires Predict Antigen-Antibody Affinity
Source: PLoS Comput Biol. 2016 Apr 13;12(4):e1004870. doi: 10.1371/journal.pcbi.1004870 (PMC4830580; doi:10.1371/journal.pcbi.1004870)
Supplement: S1 Text — Methods: Preliminary deep sequencing data analysis, multiple sequence alignment, clustering analysis, Supplementary results: Affinity predictions, structural predictions, internal contacts, ab-antigen interactions. (PDF) [file pcbi.1004870.s001.pdf]

Maximum-entropy models of sequenced immune repertoires predict antigen-antibody affinity. Supplementary Material

Lorenzo Asti[1,2], Guido Uguzzoni[2,3,4], Paolo Marcatili[5], and Andrea Pagnani[2,6]

[1]Dipartimento di Scienze di Base e Applicate per l'Ingegneria, Sapienza University of Roma, Via A. Scarpa 16, I-00161, Roma, Italy

[2]Human Genetics Foundation, Molecular Biotechnology Center, Via Nizza 52, I-10126 Torino, Italy

[3]Université Pierre et Marie Curie, UMR 7238 - Laboratoire de Génomique des Microorganismes, 15 rue de l'Ecole de Médecine, 75006 Paris, France

[4]Dipartimento di Fisica, Universià di Parma, viale G. Usberti 7/A, 43100 Parma, Italy

[5]Center for Biological Sequence Analysis, Department of Systems Biology, Technical Univeristy of Denmark, Anker Engelunds Vej, 2800 Lyngby, Denmark

[6]DISAT and Center for Computational Sciences, Politecnico Torino, Corso Duca degli Abruzzi 24, I-10129 Torino, Italy.

1 Methods

1.1 Preliminary deep sequencing data analysis

In the present Section, we outline the bioinformatic pipeline performed to extract from the raw deep sequencing data, consisting in nucleotide (nt) reads, a set of productive amino acid sequences provided with the putative IGHV and IGHJ germline genes of origin.

The raw data set contains 697079 nt reads. First the sequencing bar code has been cut away from the raw sequences. Primers (and their reverse complement) have been identified in sequences within two sequencing errors; Sequences presenting at least one primer have been maintained in the set and 3'5' sequences have been reverted and complemented. PCR bias due to different responses of the primers are expected to affect the relative abundance of sequences; Anyway, no null experiment has been performed and so there is no way to correct for these systematic errors. No further sequencing error correction scheme has been applied to nts sequences.

The resulting set of nts sequences has been analyzed with IgBLAST [8]; only sequences identified by the software as productive (i.e. that do not present a stop codon and for which the V and J genes are in frame) are kept and their amino acid (aa) translation has been considered in this work together with the first ranked IGHV and IGHJ putative germline genes of origin. Sequences presenting sequencing errors resulting in insertions and deletions whose net length is not a multiple of three are identified by IgBLAST as non-productive (V-J frame: out of frame) and they are thus automatically eliminated by this procedure.

1.2 Multiple sequence alignment

We first aligned our dataset according to the Kabat-Chothia numbering scheme using a modified version of the antibody-specific HMMs previously developed by us [3, 5]. The first modification, following the IMGT [6] and AHo [4] numbering schemes, was to place the H3 insertions symmetrically in the central position between residue 94 and 101, thus obtaining a better alignment of the H3 regions neighboring the loop stems.

As shown in Figure S1, the length of the H3 loop of antibodies in the *hypermutated cluster* varies between 7 and 26 residues, with an average length of 15.35 residues (15.25 when only considering unique sequences). There are two main peaks in the distribution, corresponding to loops of length 14 and 17. H3 length is calculated as the number of residues between position 95 and 102 included according to the Chotia numbering scheme.

The variability in the length distribution of the sequences in the *hypermutated cluster*, together with the observed sequence diversity, can be attributed to the presence of multiple clones and of intraclonal diversity.

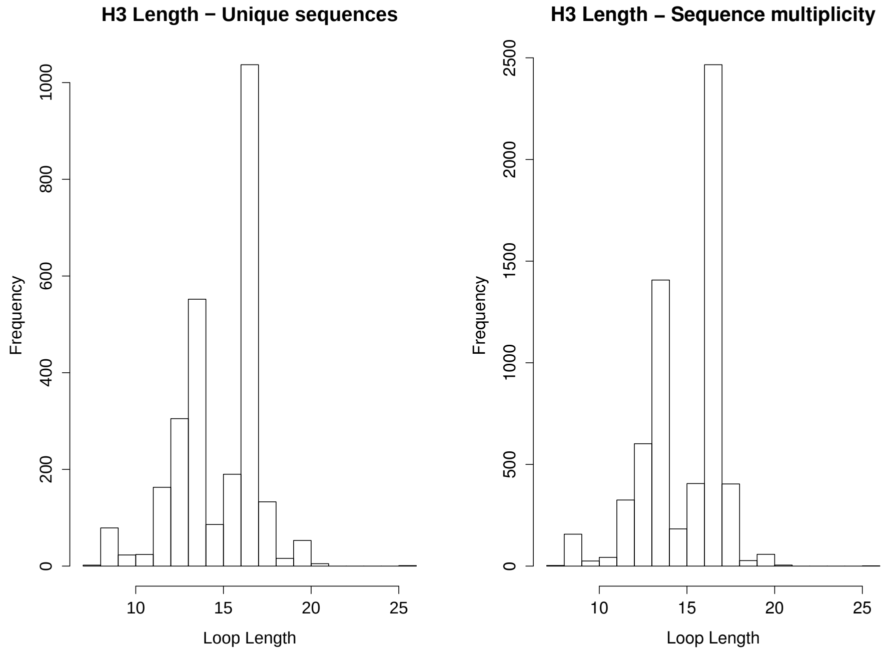

Figure S1: Left Panel: Frequency counts of the H3 loop lengths in the *hypermutated cluster*. Right Panel: Same as Left Panel but considering only unique sequences.

1.3 Clustering analysis

The set of sequences with IGHV1-2 and IGHJ2 as inferred germline gene of origin display the presence of two clearly separated clusters. This number of clusters has been confirmed by a stability analysis of the *k-means* algorithm.

To confirm the result, the set of sequences has been analyzed with the clustering algorithm proposed in [1]. This algorithm is an improved hierarchical clustering method based on the optimization of a cost function over trees of limited depth. The clustering utilizes the cavity method to find a bounded depth D spanning tree on a graph G(V, E) where V is the set of n vertices identified with the data points plus one additional root node and E is the set of edges with weights given by the dissimilarity matrix (the Hamming distance between sequences in our case) and by a unique distance λ from the root node. The external parameter λ can be used to fix the number of clusters (for $\lambda = 0$ each point is a cluster, for large enough λ all points belong to a single cluster) and to check for the structural stability of the clustering itself. Indeed, if a small increase of λ induces large decrease in the number of clusters, we take this as an indication that the structure of the clustering is weak. Robust results can be detected when for large intervals of λ values, the clusters do not change. A typical fingerprint of a robust clustering is shown in Fig. S2, where for values of $\lambda \in (50, 100)$, a stable bipartition of our data set is observed.

A direct inspection of the two clusters shows that one of the two clusters is on average more similar to the germline genes of origin (and thus named *germline cluster*), while the second cluster results to be more similar to the broadly neutralizing antibody VRC-PG04 (and named *hypermutated cluster*). The consensus sequences of the two clusters are

displayed in Fig. S3 aligned with the relative germline sequences.

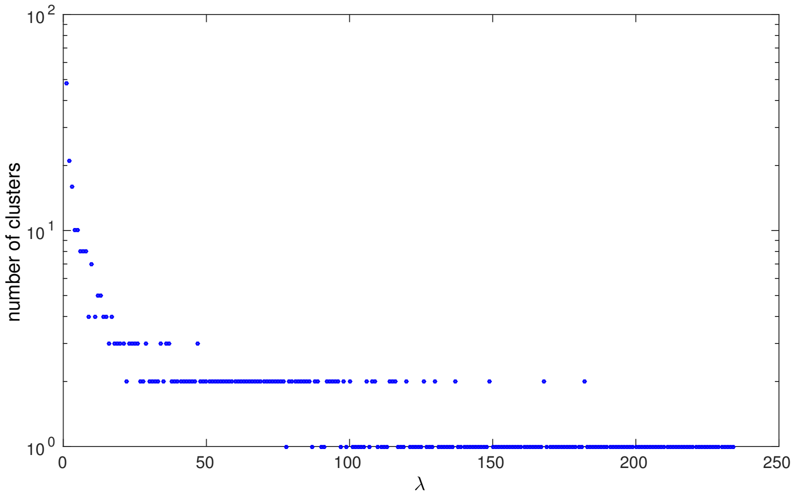

Figure S2: Number of clusters as a function of the parameter λ, see [1] for details. The long plateau at two clusters is an indication of the structural stability of the clustering.

2 Supplementary results

2.1 Affinity predictions

In Table 1 are reported for each of the 30 neutralizing Abs of the cluster PG04, the four different scores used for the affinity prediction : Multivariate Gaussian model (MGM), MGM with gap correction , MGM factorized on the alignment positions (factorized MGM), factorized MGM with gap correction .

As the donor of the sample under inspection is known to be infected with an A/D

```
>consesus_cluPG04 vs PG04:
LEQSGSGVKKPGASVRVSCWTSEDIFE-TELIHWVRQAPGQGLEWIGWVK-V-GAVNF--
| ||||||||||||||||||||||||| |||||||||||||||||||||| | |||||  
LVQSGSGVKKPGASVRVSCWTSEDIFERTELIHWVRQAPGQGLEWIGWVKTVTGAVNFGS

--FR-RVSLTRDRDLFTAHMDIRGLTQGDTATYFCARQKF----QGWYFDLWGRGTL
  || |||||||||||||||||||||||||||||||||||    |||||||||||||
PDFRQRVSLTRDRDLFTAHMDIRGLTQGDTATYFCARQKFYTGGQGWYFDLWGRGTL

>consesus_cluVJ vs germline_concatenate_IGHV1-2_e_IGHJ2:
QVQLVQSGAEVKKPGASVKVSCKASGYTF-TGYYMHWVRQAPGQGLEWMGWINPNSG
||||||||: ||||||||:||| :|   | |||||||||||||||||||||||||||
QVQLVQSGSGVKKPGASVRVSCWTSEDIFETGYYMHWVRQAPGQGLEWMGWINPNSG

GTNYAQKFQGRVTMTRDTSISTAYMELSRLRSDDTAVYYCARWYFDLWGRGTL
|||||||||||||||||||||||||||||||||||||||||||||||||||||
GTNYAQKFQGRVTMTRDTSISTAYMELSRLRSDDTAVYYCARWYFDLWGRGTL
```

Figure S3: Top: Alignment of the consensus sequence of the *hypermutated cluster* (here named "consensus_cluPG04") with the broadly neutralizing antibody VRC-PG04 sequence. Bottom: Alignment of the consensus sequence of the *germline cluster* (here named "consensus_cluVJ") with the concatenated sequences of IGHV1-2*02 and IGHJ2*01 germ-line genes.

Abs	MGM	gap-corrected MGM	factorized MGM	gap-corrected factorized MGM
9815	-1.039e+00	-1.015e+01	5.133e+01	5.838e+00
10731	-8.679e-01	-1.078e+01	5.243e+01	6.688e+00
17720	-1.161e+00	-9.685e+00	5.240e+01	6.646e+00
18278	-1.844e+00	-1.107e+01	5.011e+01	5.266e+00
24972	9.929e-02	-1.106e+01	5.570e+01	9.555e+00
31458	7.885e-01	-8.210e+00	5.346e+01	8.022e+00
43567	1.059e+00	-7.986e+00	5.382e+01	8.330e+00
47890	5.933e+00	-4.082e+00	5.574e+01	9.180e+00
53821	-2.937e-01	-9.343e+00	5.141e+01	5.988e+00
57729	2.734e+00	-6.675e+00	5.328e+01	8.207e+00
61048	-7.203e-01	-9.696e+00	5.152e+01	6.171e+00
69713	1.487e+00	-7.474e+00	5.290e+01	7.344e+00
71632	-1.747e-01	-9.313e+00	5.170e+01	6.184e+00
86277	-7.612e-01	-9.610e+00	5.138e+01	6.149e+00
86984	1.657e+00	-7.738e+00	5.331e+01	7.492e+00
95589	3.076e+00	-7.729e+00	6.011e+01	1.235e+01
96298	-1.054e+00	-1.018e+01	5.039e+01	5.642e+00
120119	2.362e+00	-6.599e+00	5.467e+01	9.241e+00
127586	3.440e+00	-5.725e+00	5.486e+01	8.996e+00
135083	-8.954e-01	-9.985e+00	5.078e+01	5.883e+00
149590	-6.527e-01	-9.639e+00	5.174e+01	6.365e+00
149768	2.709e+00	-6.426e+00	5.433e+01	8.732e+00
151901	-2.065e+00	-1.053e+01	5.236e+01	6.459e+00
164202	-1.032e+00	-9.892e+00	5.086e+01	6.366e+00
165478	-9.203e-02	-9.208e+00	5.221e+01	6.760e+00
179500	7.339e-01	-8.318e+00	5.128e+01	6.412e+00
186275	8.281e-01	-8.506e+00	5.311e+01	7.385e+00
186640	-8.288e-01	-1.013e+01	5.036e+01	5.472e+00
195462	2.679e+00	-6.730e+00	5.549e+01	9.180e+00
196147	8.306e-01	-8.783e+00	5.327e+01	7.251e+00

Table 1: MGM score of the 30 IC_{50} tested Abs. The columns refer to: antibodies code name (according to [7]), MGM score, gap-corrected MGM score, factorized MGM score, factorized gap-corrected MGM score.

recombinant HIV-1 virus, in Figure S5 we show the correlation between the MGM-score and the minimum and average neutralization power when only the 8 tested viruses that belong to clade A are considered. Coherently the predictive power of the model is even higher here, with respect to the case reported in the Main Text (see Figure S5) in which the average and minimum neutralization titers are computed over the entire set of 20 tested viruses from clade A, B and C.

In Figure S4 we show the results referred to a reduction of the horizontal size of the MSA (number of aligned residues in the sequences). By progressively eliminating columns in the MSA following their increasing entropy (i.e. variability), we observe that the Pearson correlation coefficient (i.e. the predictive power of the MGM) remains approximatively constant for a wide range of sizes, remaining essentially of the same magnitude until only the $\sim$ 60 more variable columns of the MSA are used to construct the MGM (if the correlation is computed over all the 45 tested Abs, the number of important columns reduces even to $\sim$ 10 - see Figure S4, right panel).

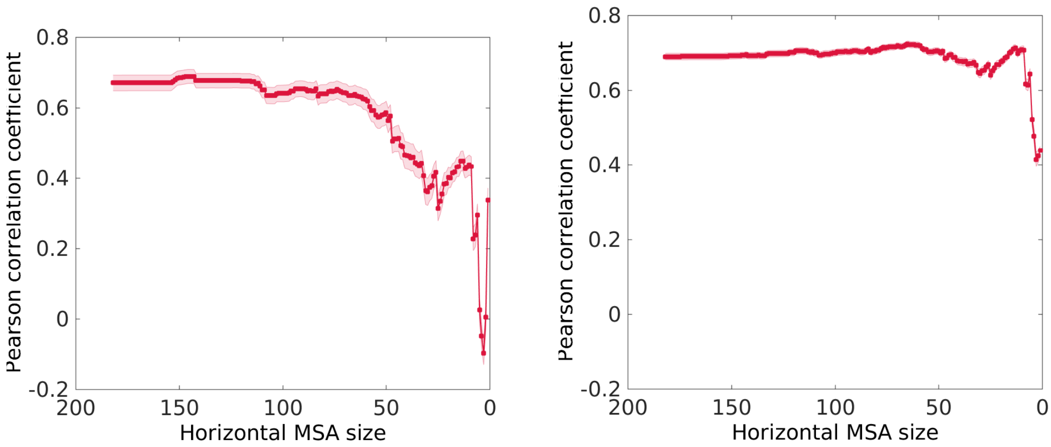

Figure S4: Iterative pruning of the least variable columns of the MSA is displayed. In both pictures, the correlation coefficient between the inferred MGM energy and the minimum neutralization titers against the tested virus isolates is displayed as the number of residues in the alignment is progressively reduced by eliminating columns according to their increasing entropy rank. The leftmost data point refers to the entire alignment. *Left panel:* The Pearson correlation coefficient is computed with the Abs belonging to the *hypermutated cluster*. The plot shows that only the first 60 more variable columns of the MSA are important for the affinity prediction. *Right panel:* the same as before for all 45 tested Abs. In this case, the number of important columns is approximately only 10.

In Figure S7 we reported the comparison between the performance of the MGM score and the score computed with pseudo-likelihood algorithm (plmDCA). The plmDCA score is less effective in reproducing affinity measurement then the MGM score but still has a better performance than the factorized MGM score and the hmm-score, as expected.

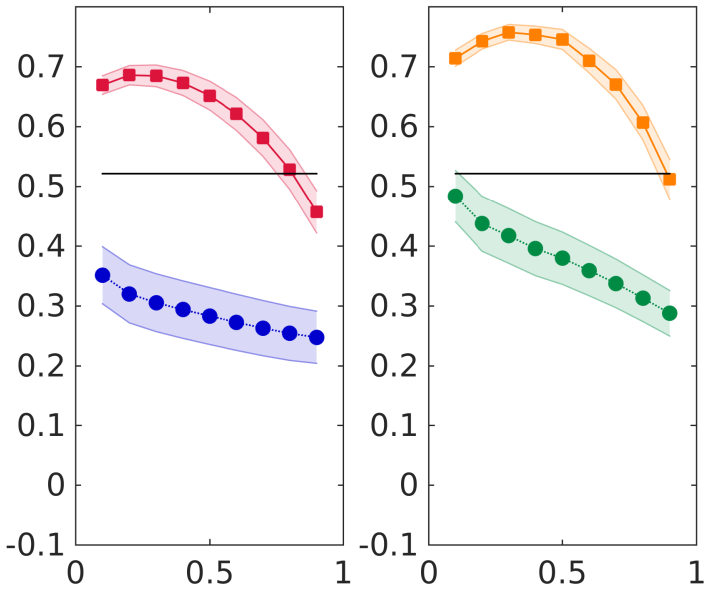

Figure S5: Pearson correlation coefficient between the inferred MGM score and the average IC_{50} neutralization titer measured over the 30 tested Abs as a function of the pseudocount parameter π (see Section Inference Methods). For each Ab, the average IC_{50} is computed **over the 8 clade A neutralized viruses** ($IC_{50} < 50\mu$g/ml). Full MGM score is represented by square bullets joined by continuous lines. Factorized MGM score is represented by circular bullets joined by dashed lines. The continuous black line shows the correlation value achieved using the hmm-score as an affinity predictor. Error bands are computed with a standard jack-knife re-sampling procedure. *Left panel*: MGM score. *Right panel*: Gap-corrected MGM score.

2.2 Structural predictions

2.2.1 Internal contacts

The gaussian DCA procedure [2] aimed at the internal contacts prediction does not give accurate results with the Rep-Seq data set under inspection. In the Main Text, the contact prediction has been performed taking as input the set of sequences belonging to the *hypermutated cluster*. Here we report the result of the contact inference over the whole Rep-Seq data set. Even if the enlargement of the set of sequence slightly increases the performances, the predictive power is still unsatisfactory as shown in Figure S8.

2.2.2 Ab-antigen interactions

In this Section, we present the supplementary results referred to a reduction of the horizontal size of the MSA (number of aligned residues in the sequences). By progressively eliminating columns in the MSA following their decreasing entropy (i.e. variability) rank some structural information on the Ab-antigen interaction mode is recovered.

In fact, as columns columns are removed starting from the more variable to the more constant, the reduction of the predictive power is fast and, in particular, the decreasing is more pronounced in correspondence of the deletion of a few residues that are highlighted

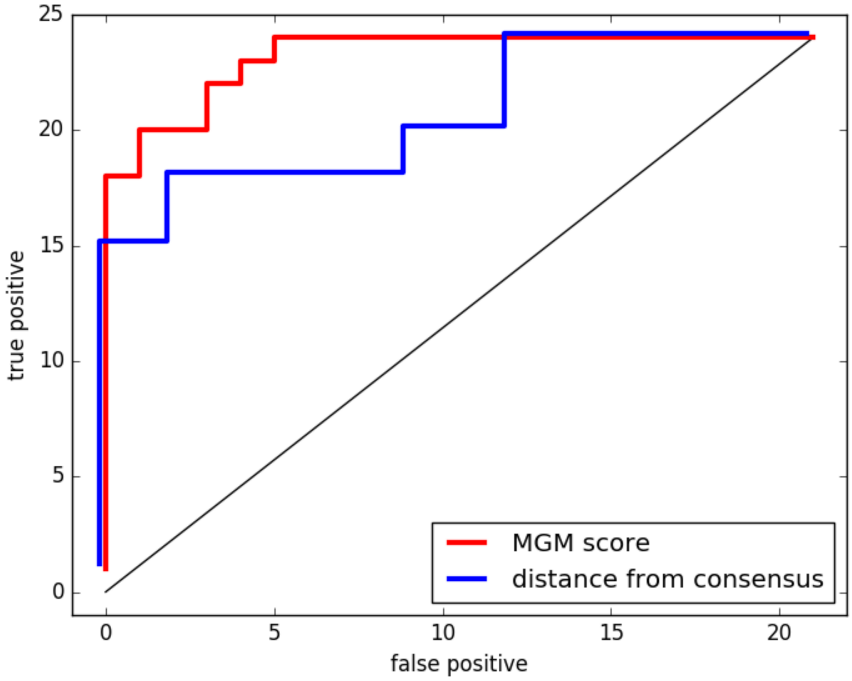

Figure S6: ROC curve testing the performance of the MGM-score (red line) in discriminating binding vs. non-binding sequences with a (normalized) area under the ROC = 0.97. Using as a simpler score function the distance from the consensus sequence of the hypermutated cluster (blue line), we obtain a (normalized) area under the ROC of 0.86.

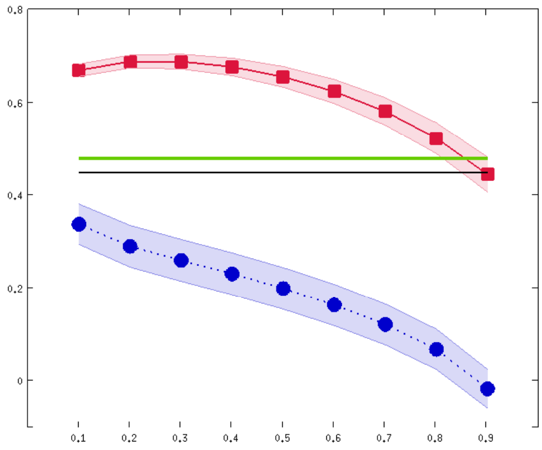

Figure S7: Comparison of the performance of the MGM score and the score computed with the pseudo-likelihood algorithm (plmDCA). The Pearson correlation coefficient between the inferred MGM score and the average IC_{50} neutralization titer measured over the 30 tested Abs as a function of the pseudocount parameter π (see Section Inference Methods). For the plmDCA are reported the Pearson correlation value of the best performance model over the optimization of the regularization parameters. For each Ab, the average IC_{50} is computed over the viruses. Full MGM score is represented by square bullets joined by continuous lines. Factorized MGM score is represented by circular bullets joined by dashed lines. The green continuous line shows the correlation value using plmDCA score. The continuous black line shows the correlation value achieved using the hmm-score as an affinity predictor. Error bands are computed with a standard jack-knife re-sampling procedure.

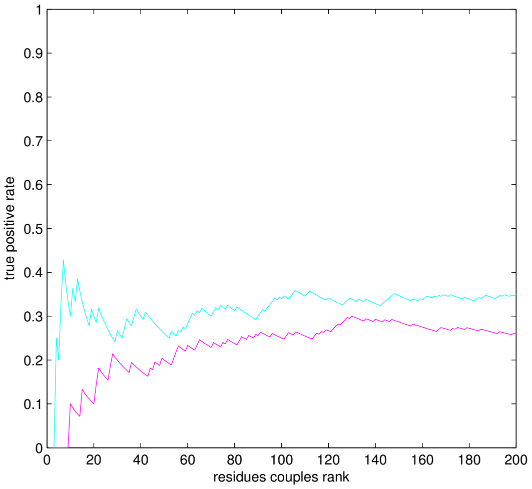

Figure S8: Contact prediction true positive rate of Mutual Information (pink) and Direct Information (light blue) analysis performed on the whole set of productive sequences. The reweighting parameter (see [2]) as been set to 0.01 in this case. Two residues are considered to be in contact if at least a couple of atom is at distance lower than 8 Å.

with arrows in Figure S9. As discussed in the Main Text (Section 3.1 and Table 2), these residues are often observed to play a structural role in the antigen recognition. This kind of analysis shows how structural information on the antigen recognition mode can in principle be retrieved in cases in which the tridimensional structure of the Ab-antigen complex is lacking but Rep-Seq data and affinity measurements are available.

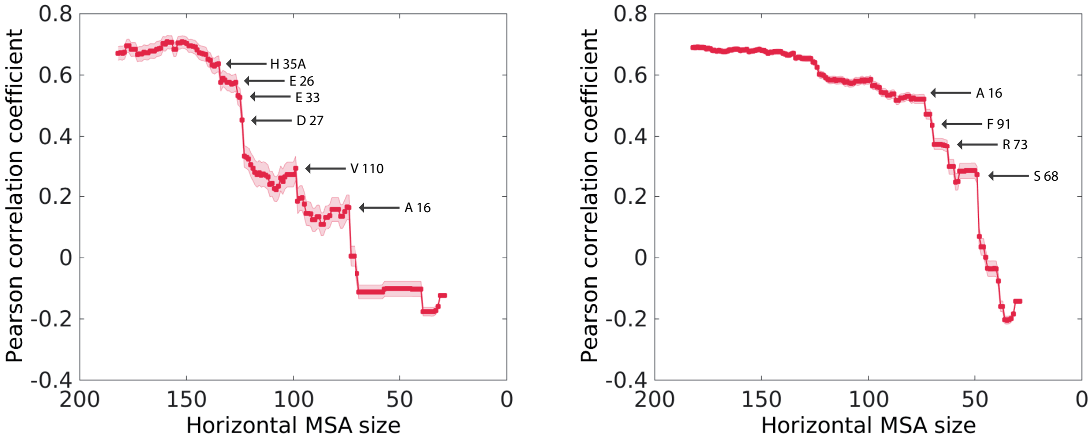

Figure S9: Iterative pruning of the most variable columns of the MSA is displayed. In both pictures, the correlation coefficient between the inferred MGM energy and the minimum neutralization titers against the tested virus isolates is displayed as the number of residues in the alignment is progressively reduced by eliminating columns according to decreasing entropy rank. The leftmost data point refers to the entire alignment. Residues whose deletion causes a remarkable decreasing of the correlation are highlighted. *Left panel:* the Pearson correlation coefficient is computed with the Abs belonging to the *hypermutated cluster*. *Right panel:* all 45 experimentally tested Abs are considered.

References

[1] M. Bailly-Bechet, S. Bradde, A. Braunstein, A. Flaxman, L. Foini, and R. Zecchina. Clustering with shallow trees. *Journal of Statistical Mechanics: Theory and Experiment*, 2009(12):P12010, 2009.

[2] Carlo Baldassi, Marco Zamparo, Christoph Feinauer, Andrea Procaccini, Riccardo Zecchina, Martin Weigt, and Andrea Pagnani. Fast and accurate multivariate gaussian modeling of protein families: Predicting residue contacts and protein-interaction partners. *PLoS ONE*, 9(3):e92721, 2014.

[3] Anna Chailyan, Anna Tramontano, and Paolo Marcatili. A database of immunoglobulins with integrated tools: Digit. *Nucleic acids research*, page gkr806, 2011.

[4] Annemarie Honegger and Andreas PluÈckthun. Yet another numbering scheme for immunoglobulin variable domains: an automatic modeling and analysis tool. *Journal of molecular biology*, 309(3):657–670, 2001.

[5] Paolo Marcatili, Pier Paolo Olimpieri, Anna Chailyan, and Anna Tramontano. Antibody modeling using the prediction of immunoglobulin structure (pigs) web server. *Nature Protocols*, 9(12):2771–2783, 2014.

[6] Hiroki Shirai, Catherine Prades, Randi Vita, Paolo Marcatili, Bojana Popovic, Jianqing Xu, John P Overington, Kazunori Hirayama, Shinji Soga, Kazuhisa Tsunoyama, et al. Antibody informatics for drug discovery. *Biochimica et Biophysica Acta (BBA)-Proteins and Proteomics*, 1844(11):2002–2015, 2014.

[7] Xueling Wu, Tongqing Zhou, Jiang Zhu, Baoshan Zhang, Ivelin Georgiev, Charlene Wang, Xuejun Chen, Nancy S Longo, Mark Louder, Krisha McKee, et al. Focused evolution of hiv-1 neutralizing antibodies revealed by structures and deep sequencing. *Science*, 333(6049):1593–1602, 2011.

[8] Jian Ye, Ning Ma, Thomas L Madden, and James M Ostell. Igblast: an immunoglobulin variable domain sequence analysis tool. *Nucleic acids research*, page gkt382, 2013.
